# Supplementary material for: Adsorption and Photodegradation of Lanasol Yellow 4G in Aqueous Solution by Natural Zeolite Treated by CO2-Laser Radiation
Source: Materials (Basel). 2023 Jul 6;16(13):4855. doi: 10.3390/ma16134855 (PMC10343540; doi:10.3390/ma16134855)
Supplement: Supplementary file 1 [file materials-16-04855-s001.zip › materials-2448176-supplementary.pdf]

Supplementary Material

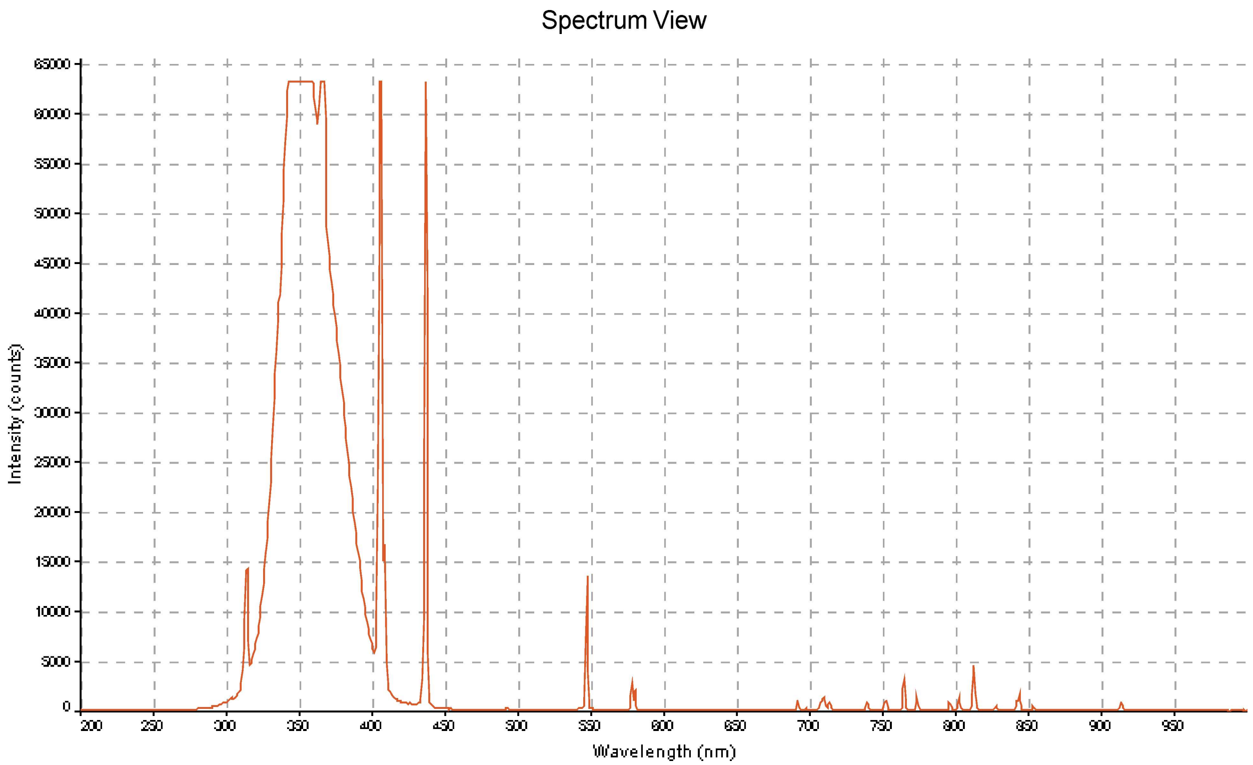

Figure S1. Emission spectra of the UV-A lamp used in the photodegradation process.
